# A Study of FoxO1, mTOR, miR-21, miR-29b, and miR-98 Expression Levels Regarding Metabolic Syndrome in Acne Vulgaris Patients

**DOI:** 10.7759/cureus.56562

**Published:** 2024-03-20

**Authors:** Nazan Akdağ, Engin Atli, Drenushe Zhuri̇, Hazal Sezgi̇ner Güler, Yıldız Gürsel Ürün

**Affiliations:** 1 Department of Dermatology and Venereology, Trakya University Faculty of Medicine, Edirne, TUR; 2 Department of Medical Genetics, Trakya University Faculty of Medicine, Edirne, TUR

**Keywords:** acne vulgaris, metabolic syndrome, forkhead box protein, mechanistic target of rapamycin, microrna (mirna)

## Abstract

Background: Acne vulgaris (AV) is an inflammatory skin disease caused by the mechanistic target of rapamycin complex 1 (mTORC1). forkhead box protein (Fox) O1 is known to regulate the relationship between the mTORC1 signaling pathway and insulin resistance (IR). Increased mTORC1 signaling is known to predispose one to diseases such as insulin resistance (IR), obesity, and diabetes mellitus. One of the major components of mTORC1 is mTOR. FoxO1 and mTOR play key roles in the onset and progression of metabolic syndrome (MetS). In this study, we aimed to elucidate the relationship between AV and MetS through FoxO1 and mTOR signaling pathways and microRNAs (miRs) associated with these signaling pathways.

Methods: We examined 20 AV patients without MetS, 16 AV patients with MetS, and 20 healthy controls. The demographic characteristics of the patients, MetS parameters, clinical severity of AV (Global Acne Grading System, GAGS), and the homeostasis model assessment (HOMA) values were compared between the groups. In addition, the expression levels of FoxO1 and mTOR genes, along with the expression levels of miR-21, miR-29b, and miR-98, were assessed in skin biopsy samples from all groups using real-time polymerase chain reaction methods. FoxO1, mTOR, and miRNA expression levels were recorded as fold change.

Results: The mean age of patients with AV without MetS was statistically lower. In AV patients with MetS, those with moderate GAGS scores had statistically significantly higher HOMA values than those with mild GAGS scores. FoxO1 expression was significantly lower in AV patients compared to controls. The mTOR expression levels of AV patients with MetS were significantly higher than the other two groups. The expression levels of miR-21 and miR-29b were significantly increased in the group of AV patients with MetS compared to the group of AV patients without MetS.

Conclusions: These results suggested that the mTOR pathway may play an important role in explaining the relationship between AV and MetS in acne pathogenesis. They also suggested that miR-21 and miR-29b play a role in the inflammatory process of AV.

## Introduction

Acne vulgaris (AV) is a chronic and inflammatory condition affecting the pilosebaceous unit. It has a multifactorial etiology [1,2]. The main etiopathogenic factors of AV include follicular hyperkeratinization, increased sebum production, and increased bacterial colonization (Propionibacterium acnes) of the pilosebaceous unit. The consequence of these factors is an increase in the release of pro-inflammatory cytokines. Insulin/insulin-like growth factor 1 (IGF-1) plays a role in maintaining this inflammatory process [3].

Metabolic syndrome (MetS) is defined as a syndrome of chronic inflammation with persistently elevated pro-inflammatory cytokine levels [4]. IR is recognized as a component of MetS [5]. In an experimental study, insulin resistance (IR) was found to cause or exacerbate AV [6]. High levels of pro-inflammatory cytokines induced by IR alter the balance of proliferation and differentiation in keratinocytes, leading to increased chronic inflammation and oxidative stress [4].

Increased insulin/IGF-1 signaling leads to the activation of the phosphatidyl inositol 3-kinase (PI3K)-protein kinase B (Akt) pathway (PI3K-Akt). The activation of PI3K-Akt leads to the phosphorylation of forkhead box protein O1 (FoxO1) in the nucleus and the excretion of FoxO1 into the cytoplasm through the loss of its transcriptional activity [7]. The inhibition of FoxO1 results in the activation of the mechanistic target of rapamycin signaling complex 1 (mTORC1) [8]. It is known that mTORC1 activity is increased in patients with AV and the increased mTORC1 signaling is associated with the emergence of several diseases, including IR, obesity, and diabetes mellitus [9]. One of the major components of mTORC1 is the mechanistic target of rapamycin (mTOR). mTOR forms the catalytic subunit of the protein complexes mTORC1 and mTORC2. mTOR is a nutrient-sensitive regulator of cell growth and proliferation, lipid synthesis, and protein translation [8].

MicroRNAs (miRs) are small RNA molecules transcribed from RNA genes in protein-coding and non-protein-coding regions of the genome but are not translated into proteins [10, 11]. miRs affect keratinocyte maturation and cooperation and participate in the pathogenesis of AV, resulting in different outcomes depending on the deficiency or overexpression of their levels [12].

miR-21 plays a role in AV pathogenesis by increasing PI3K-AKT signaling by decreasing the nuclear activity of FoxO1 [13]. The miR-29 family targets genes and transcription factors related to the signaling pathways of insulin and glucose metabolism, mTOR, and thyroid hormones that regulate energy homeostasis [14]. miR-98 is known to regulate both glucose and lipid metabolism in diabetic patients [15].

The aim of this study is to compare the expression levels of FoxO1 and mTOR genes in skin biopsies from AV patients with and without MetS and to investigate their epigenetic reflection. To this end, the changes in the expression levels of miR-21, miR-29b, and miR-98 acting through these pathways in the tissues of patients with AV and healthy subjects will be discussed. Therefore, we sought to explore the effects of MetS on AV pathogenesis.

## Materials and methods

Sample selection

Fifty-six patients participated in this study: 20 patients with AV without MetS, 16 patients with AV with MetS, and 20 healthy control patients. The National Cholesterol Education Program ATP III diagnostic criteria were used to diagnose MetS [16]. The Global Acne Grading System (GAGS) was used to evaluate the severity of acne in patients with AV [17]. Patients with systemic diseases or skin conditions other than acne were not included in the study. Patients who received topical acne treatment for at least two weeks, did not take systemic antibiotics for at least four weeks, and took oral isotretinoin for at least six months were included in the study [1]. This study was conducted at Trakya University Faculty of Medicine, Edirne, Turkey. The study was approved by the Scientific Research Ethics Committee of the Dean of the Trakya University Faculty of Medicine (protocol code: TUFM-SREC 2020/37; approval date: January 20, 2020). Patients were informed of the details and potential risks of the study, and a signed informed consent form was obtained prior to enrollment.

Study design

The demographic data and clinical characteristics (gender, age, smoking and alcohol consumption, family history, height, weight, body mass index (BMI), abdominal circumference, and systolic/diastolic blood pressure) of the patients and healthy controls who consented to participate in the study were recorded. After a minimum eight-hour fasting night, 10 ml of peripheral venous blood was drawn, and fasting plasma glucose, fasting plasma insulin, triglycerides (TGs), and high-density lipoprotein cholesterol (HDL-C) levels of each participant were measured (Roche c702 and e801 analyzers). Systolic and diastolic blood pressures were measured ten minutes after a five-minute rest period. The homeostasis model assessment (HOMA) index was calculated for all patients [5]. 

Skin biopsies were obtained with a 4-mm punch biopsy (Kai® Medical, Seki City, Gifu, Japan) from the region where lesions were most severe in the patients with AV and from the back regions of the healthy control subjects. Biopsy samples were stored at -80° in RNAlater stabilization solution (Thermo Fisher Scientific, Inc., Waltham, Massachusetts, United States) until RNA isolation.

Selection of FoxO1 and mTOR

FoxO1 and mTOR genes were included in the study by performing gene ontology analysis using databases such as the Kyoto Encyclopedia of Genes and Genomes (KEGG) pathway [18], pathway unification database (PathCards) [19], and the literature. 

Selection of miRs

Using the miR sequence database (https://www.mirbase.org/) [20] and literature searches, miR-21 was shown to be among the many potential targets. In the miRBase, miR-29b was found to be the mTOR target gene miRBase accession for hsa-miR-29b-2 [20]. A search of the miRBase and the molecular signatures database (https://www.gsea-msigdb.org/gsea/msigdb/) showed that FoxO1 is one of the target genes associated with miR-98 [20,21].

Gene expression and miR analysis

In the Medical Genetics Laboratory, we utilized the Pure Link® RNA Mini Kit (Thermo Fisher Scientific, Inc., Waltham, Massachusetts, United States) to isolate RNA from tissue samples collected from both the patient and control groups, following the manufacturer's guidelines. The High-Capacity cDNA Reverse Transcription Kit (Thermo Fisher Scientific, Inc., Waltham, Massachusetts, United States) was used to convert RNA samples into complementary deoxyribonucleic acid (cDNA). miR expression analyses were performed by real-time polymerase chain reaction (real-time PCR) using the TaqMan Advanced miR Assay (Thermo Fisher Scientific, Inc., Waltham, Massachusetts, United States). Gene expression analyses were performed by real-time PCR using the protocol of the TaqMan gene expression assay kit. To determine the expression levels of our target genes included in the study (FoxO1 and mTOR), the ACTINβ gene was used as a housekeeping gene. Similarly, the U6 reference miR was used for the miR expression study. In the study, gene and miR samples were repeated three times for each patient and healthy subject. First, ΔCt values were calculated from the obtained data. For gene expression studies, the ΔΔCt values were calculated first, followed by the 2-ΔΔCt values, and the results were statistically analyzed. According to the data obtained from the study, miRs and gene expression levels that showed a change (increase or decrease) of two-fold or more in terms of expression levels were considered significant. 

Statistical analysis

Statistical analyses of the data were performed using the program IBM SPSS Statistics for Windows, Version 24, (Released 2016; IBM Corp., Armonk, New York, United States). Comparisons of categorical data between groups were conducted using Fisher's exact test and Pearson's chi-square test. Because continuous data lacked normal distribution properties, the Mann-Whitney U test was used for comparison between two groups, and Kruskal-Wallis H statistical analysis (Mann-Whitney U test with postoperative Bonferroni correction) was used for comparison between more than two groups. When a difference was detected between the groups using the Kruskal-Wallis test, a post hoc analysis of subgroups was performed to determine which groups exhibited differences. A p-value < 0.05 was considered statistically significant.

## Results

When the sociodemographic distribution of the 20 patients with AV without MetS, the 16 patients with AV with MetS, and the 20 healthy control patients was examined, a statistically similar distribution was found with respect to characteristics such as gender, tobacco smoking, alcohol consumption, and family history of acne. It was found that the mean age of the patients with AV without MetS was statistically lower (p = 0.001). The mean age of the 56 patients included in the study was 27.38±6.22 years. A total of 73.2% (n = 41) of the patients were male (Table 1).

**Table 1:** Comparison of patients' demographic characteristics (n = 56) ^*^Fisher’s exact test, ^†^Kruskal-Wallis H analysis, ^‡^Pearson’s Chi-square test. Min: Minimum; Max: Maximum

Demographic characteristics	Controls	Metabolic syndrome: positive	Metabolic syndrome: negative	X^2^	p-value
	n (%)	n (%)	n (%)		
Gender					
Male	13 (65)	11 (68.8)	17 (85)	2.324	0.402^*^
Female	7 (35)	5 (31.3)	3 (15)		
Age					
Median (min-max)	27.5 (23-43)	27 (20-41)	22.5(20-45)	13.684	0.001^†^
Tobacco smoking					
No	14 (70)	8 (50)	7 (35)	4.935	0.085^‡^
Yes	6 (30)	8 (50)	13 (65)		
Alcohol consumption					
No	12 (60)	4 (25)	8 (40)	4.55	0.103^‡^
Yes	8 (40)	12 (75)	12 (60)		
Family history					
No	13 (65)	10 (62.5)	15 (75)	0.753	0.686^‡^
Yes	7 (35)	6 (37.5)	5 (25)		

BMI, abdominal circumference (cm), systolic/diastolic blood pressure (mmHg), and laboratory values were analyzed between groups. According to the results, BMI, abdominal circumference, systolic and diastolic blood pressures, fasting plasma insulin and TGs ratios, and HOMA values were higher in AV patients with MetS than in AV patients without MetS and the healthy control group, as expected (p < 0.05). The plasma HDL-C levels of the patients with MetS were statistically significantly lower than those of the AV patients without MetS and the healthy control group (p = 0.028, Table 2).

**Table 2:** Distribution of the 56 patients according to BMI, abdominal circumference, systolic/diastolic blood pressure, and laboratory exams ^*^Kruskal-Wallis H analysis. BMI: Body mass index; HOMA: Homeostatic model assessment; TGs: Triglycerides; HDL-C: High-density lipoprotein cholesterol; Min: Minimum; Max: Maximum

Parameters	Controls		Metabolic syndrome: positive		Metabolic syndrome: negative		x^2^	p-value^*^
	Median	(min-max)	Median	(min-max)	Median	(min-max)		
BMI (kg/m^2^)	22.99	(20.28-29.22)	30.11	(23.94-35.56)	23.75	(19.32-29.22)	25.181	0.000
Abdominal circumference (cm)	86	(58-116)	104.5	(92-145)	88	(65-146)	19.034	0.000
Systolic blood pressure (mmHg)	120	(110-150)	140	(110-170)	130	(90-140)	9.130	0.010
Diastolic blood pressure (mmHg)	80	(60-85)	87.5	(80-100)	80	(65-90)	16.662	0.000
Fasting plasma glucose (mg/dL)	88	(79-123)	93	(74-116)	88.5	(73-108)	1.388	0.500
Fasting plasma insulin (μIU/mL)	5.5	(1.7-44)	8.75	(4.9-64)	4.32	(1.1-25)	10.028	0.007
HOMA	1.2	(0.4-13.4)	2.05	(0.3-18.3)	0.95	(0.2-5.8)	7.717	0.021
Plasma TGs (mg/dl)	104.5	(35-414)	179	(67-485)	111.5	(47-252)	12.514	0.002
Plasma HDL-C (mg/dl)	50.5	(34-73)	40.5	(28-60)	51.5	(30-86)	7.164	0.028

Looking at the GAGS score, 22.2% (n = 8) had mild AV, and 55.6% (n = 20) of the patients had moderate AV. When the distribution of acne severity was examined according to the presence of MetS, no statistically significant difference was found between the groups (Table 3).

**Table 3:** Distribution of acne vulgaris severity in patients according to the presence of metabolic syndrome ^*^Severity is classified as mild when the score is 1-18, moderate when the score is 19-30, severe when the score is 31-38, and very severe when the score is more than 38, following the author's recommendation [17]. ^†^Fisher's exact test. GAGS: Global Acne Grading System

Acne vulgaris severity (based on GAGS score)^*^	Metabolic syndrome: positive n (%)	Metabolic syndrome: negative n (%)	Total n (%)	x^2^	p-value^†^
Mild	5 (31.3)	3 (15.0)	8 (22.2)	2.601	0.564
Moderate	8 (50.0)	12 (60.0)	20 (55.6)		
Severe	3 (18.8)	3 (15.0)	6 (16.7)		
Very severe	0 (0.0)	2 (10.0)	2 (5.6)		
Total	16 (44.4)	20 (56.6)	36 (100.0)		

In AV patients with MetS, a statistically significant difference was observed between the groups in terms of HOMA values according to GAGS scores (p = 0.027). When attempting to determine from which group the difference originated, it was discovered that the HOMA values of the patients in the group with moderate GAGS score were statistically significant compared to those in the group with mild GAGS score (p < 0.0167). In AV patients without MetS, when GAGS scores were evaluated between the groups in terms of HOMA values in all cases, no statistically significant difference was detected (p > 0.05) (Table 4). 

**Table 4:** Distribution of acne vulgaris severity in patients according to the presence of HOMA ^*^Severity is classified as mild when the score is 1-18, moderate when the score is 19-30, severe when the score is 31-38, and very severe when the score is more than 38, following the author's recommendation [17]. ^†^Kruskal-Wallis H analysis. HOMA: Homeostasis model assessment; GAGS: Global Acne Grading System; SD: Standard deviation; Min: Minimum; Max: Maximum

	GAGS^*^	HOMA		x^2^	p- value^†^
		Mean±SD	Median (min-max)		
Acne vulgaris patients with metabolic syndrome	Mild	1.46±0.78	1.35 (0.3-2.6)	7.209	0.027
	Moderate	9.86±7.64	13.4 (1.6-18.3)		
	Severe	5.5±3.01	6.2 (2.2-8.1)		
Acne vulgaris patients without metabolic syndrome	Mild	1.77±2.03	0.8 (0.4-4.1)	0.750	0.861
	Moderate	1.58±1.73	0.9 (0.2-5.8)		
	Severe	1.1±0.87	0.6 (0.6-2.1)		
	Very severe	1.55±0.07	1.55 (1.5-1.6)		

**Figure 1:**
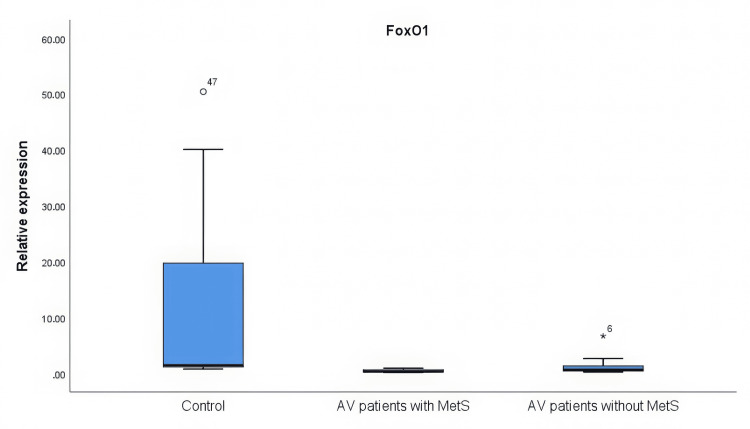
FoxO1 gene expression levels in AV patients and control individuals It was determined that the FoxO1 expression level decreased in both AV patients with MetS and AV patients without MetS when compared to the control group (p = 0.000). The statistical analysis was performed by the Kruskal-Wallis test. AV: Acne vulgaris; MetS: Metabolic syndrome; FoxO1: Forkhead box protein O1

**Figure 2:**
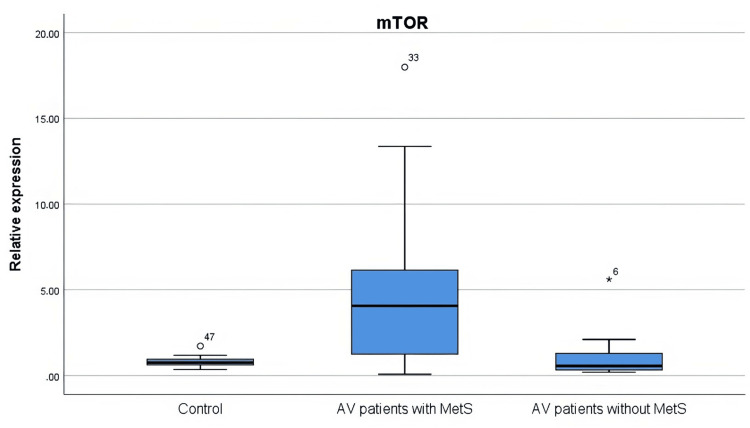
mTOR gene expression levels in AV patients and control individuals mTOR gene expression levels were increased in AV patients with MetS compared to the control group (p = 0.011) and in AV patients without MetS (p = 0.012). The statistical analysis was performed by the Kruskal-Wallis test. AV: Acne vulgaris; MetS: Metabolic syndrome; mTOR: Mechanistic target of rapamycin

The expression levels of FoxO1 and mTOR were investigated in the tissues of the healthy controls and the patients with AV. It was found that FoxO1 expression was significantly lower in the AV patients compared to the controls (p = 0.000). The AV patients with MetS demonstrated significantly elevated mTOR expression levels compared to both the control group (p = 0.011) and the AV patients without MetS (p = 0.012) (Figures 1, 2).

**Figure 3:**
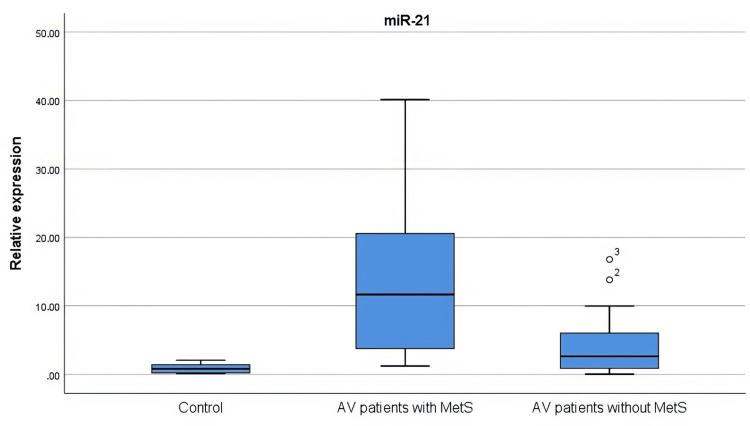
miR-21 levels in AV patients with and without MetS and controls miR-21 levels were higher in both AV patients with MetS (p = 0.000) and AV patients without MetS (p = 0.005) compared to the control group. The statistical analysis was performed by the Kruskal-Wallis test. AV: Acne vulgaris; MetS: Metabolic syndrome; miR: microRNA

**Figure 4:**
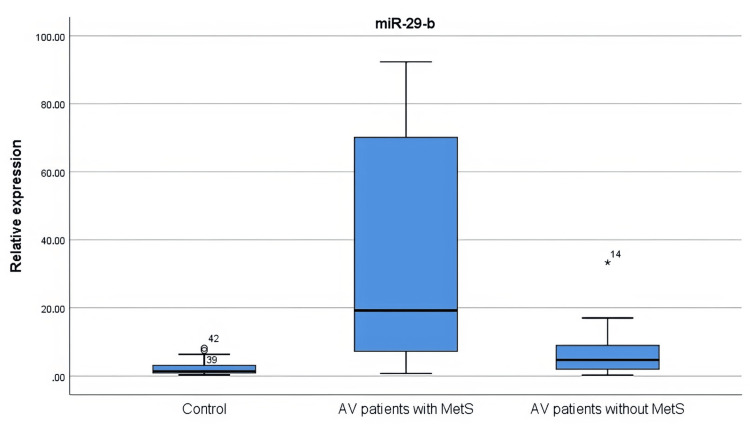
miR-29b levels in AV patients with and without MetS and controls There was a significant increase in miR-29b in both AV patients with MetS (p = 0.000) and AV patients without MetS (p = 0.016) compared to the control group. The statistical analysis was performed by the Kruskal-Wallis test. AV: Acne vulgaris; MetS: Metabolic syndrome; miR: microRNA

**Figure 5:**
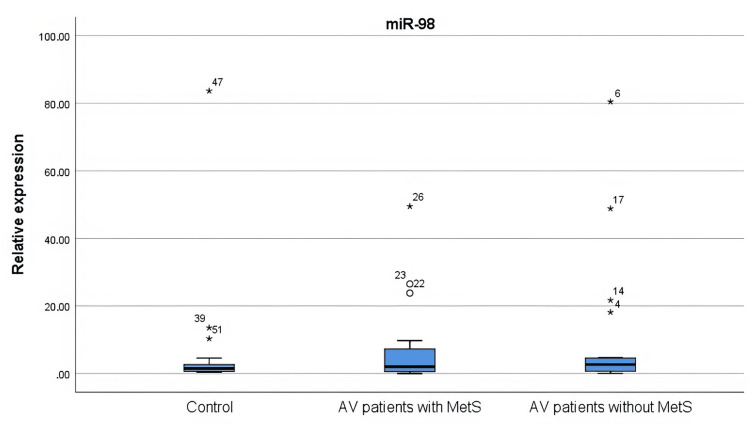
miR-98 levels in AV patients with and without MetS and controls There was no statistical difference in miR-98 expression levels in all three groups. The statistical analysis was performed by the Kruskal-Wallis test. AV: Acne vulgaris; MetS: Metabolic syndrome; miR: microRNA

The expression levels of miR-21, miR-29b, and miR-98 in the tissues of the healthy controls and the patients with AV were examined. One patient with a statistically high standard deviation of miR-21 expression level (5330.3) was not included in the analysis. The miR-21 level was higher in both the AV patients with MetS (p = 0.000) and the AV patients without MetS (p = 0.005) compared to the control group. miR-29b was higher in both the AV patients with MetS (p = 0.000) and the AV patients without MetS (p = 0.016) compared to the control group. It was observed that the expression levels of miR-21 and miR-29b were significantly increased in the group of AV patients with MetS compared to the group of AV patients without MetS (p = 0.001 and p = 0.005, respectively). The expression level of miR-98 showed a similar distribution between groups (Figures 3-5). 

## Discussion

Due to its design, our study had differences in age and gender. Physiological IR, which begins at puberty and continues into the 20s, leads to hyperinsulinemia, and hyperinsulinemia leads to increased androgen synthesis and paves the way for the development of AV [22]. The occurrence of acne after the age of 20 is due to both endogenous and exogenous factors [23]. Our study included patients over 20 years of age, so our patients were not affected by physiological IR, and the mean age of patients with AV was 27.38 years. The prevalence of MetS increases with age [3,24]. Our study findings indicated that patients with AV and MetS had a higher mean age compared to patients with AV and no MetS, which aligns with the existing literature on the topic.

While the prevalence of AV in adolescents is quite similar in both sexes, acne in adulthood occurs more frequently in women than in men [23]. In our study, the male population dominated. In a cross-sectional study comparing the metabolic condition of 100 male acne patients and 100 healthy male control subjects, the prevalence of MetS was 17% in acne patients and 9% in control subjects. It was also concluded that the prevalence of IR was significantly higher in acne patients (22%) than in control subjects (11%) [22]. Stefanadi et al. [23] found an increased prevalence of IR in male acne patients compared to controls and recommended that these patients be regularly screened for hyperinsulinemic disease because of the risk of developing type 2 diabetes mellitus. The high proportion of male patients with AV in our study may be related to the inclusion of patients with MetS and increased IR in male patients.

In the literature, there are various data available regarding the relationship between MetS and AV. Melnik [9] has considered that sebaceous follicles represent MetS. In a new study published in 2023, it was reported that MetS incidence was higher in patients with adult AV, and there was a positive correlation between AV severity and MetS [24]. In our study, no association was found between the severity of AV and MetS. We suspect that this was due to the small study population.

Homeostatic model assessment is a safe way to determine IR [25]. Del Prete et al. [26] demonstrated that MetS components are associated with the occurrence of acne in male AV patients, and they specifically focused on the parallelism of HOMA values with acne severity. When our study and the results from the literature are evaluated together, high HOMA values could be related to the severity of acne. 

**Figure 6:**
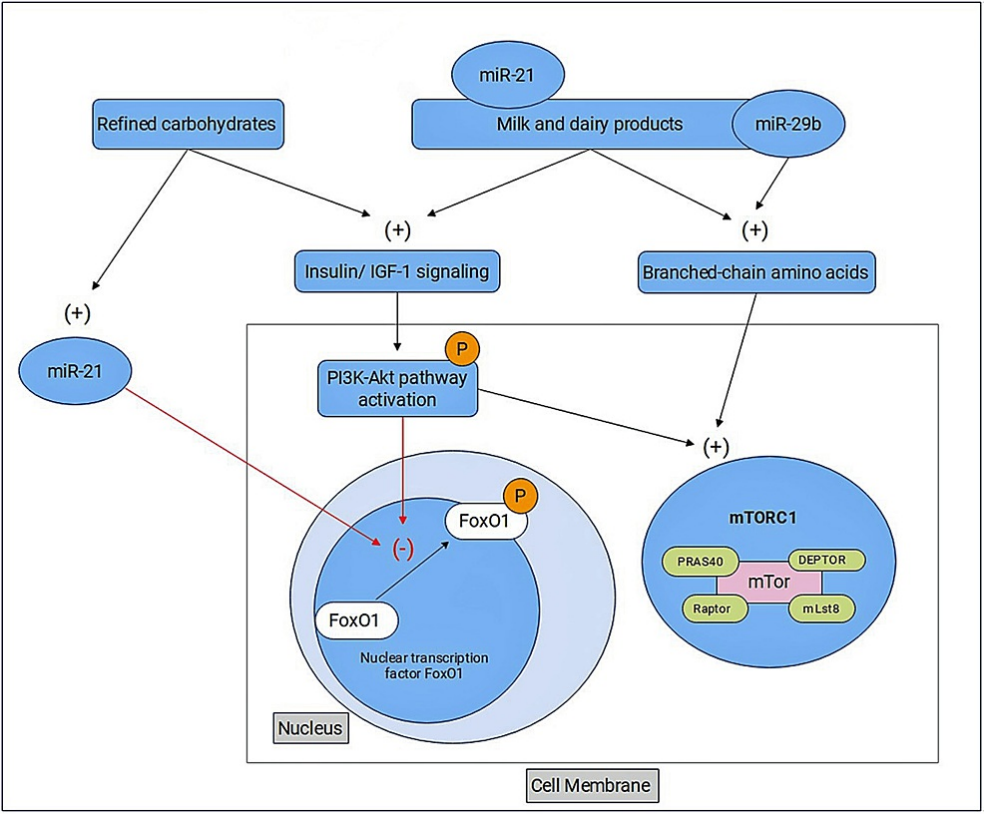
The relationship between FoxO1, mTOR, miR-21, and miR-29b in the pathogenesis of acne vulgaris Adapted from Melnik’s articles [9,27]. IGF-1: Insulin-like growth factor-1; FoxO1: Forkhead box protein O1; miR: microRNA; PI3K-Akt: Phosphatidyl inositol 3-kinase (PI3K)-protein kinase B (Akt); mTORC1: Target of rapamycin signaling complex 1; PRAS40: The proline-rich Akt substrate, 40 kDa; DEPTOR: DEP domain containing mTOR interacting protein; mTOR: Mechanistic target of rapamycin; Raptor: Regulatory associated protein of mTOR; mLst8: Mammalian LST8 homolog

The increase in insulin/IGF-1 levels impairs FoxO1-mediated gene regulation in patients with AV, resulting in FoxO1 inhibition by nuclear extrusion (Figure 6) [26,27]. 

In the study by Agamia et al. [8], the nuclear transcription factor FoxO1 was determined to be significantly increased in patients with severe acne by stimulating IGF-1-mediated transfer from the nucleus to the cytoplasm. Thus, a significant deficiency was detected. Lembo et al. [28] reported that they did not detect a significant increase in the expression level of FoxO1 in the tissues of AV and psoriasis patients compared to healthy tissues. In our study, a significant decrease in FoxO1 gene expression was detected in the tissues of AV patients compared with control subjects. This provides additional evidence of the involvement of the FoxO1 gene in the pathogenesis of AV.

Recently, studies have focused on the PI3K/Akt/mTOR axis in the pathogenesis of AV (Figure 6). In the study by Agami et al. [8], it was found that the mTOR pathway is involved in the development of acne. The researchers mentioned that mTOR expression is increased in immunohistochemical studies in patients with acne at both the nuclear and cytoplasmic levels. Lembo et al. [28] concluded that mTOR expression levels were increased in AV and psoriasis patients. Nevertheless, while mTOR fold increase did not correlate with FoxO1 in psoriasis patients, a significant correlation was found between mTOR and FoxO1 in AV patients. Monfrecola et al. [29] determined a significant increase in mTOR expression level in lesional and nonlesional skin biopsies from acne patients compared with healthy tissue. The results of our study support the literature. The mTOR expression is increased in patients with AV.

Theoretically, increased insulin/IGF-1 levels would be expected to lead to greater suppression of FoxO1 expression in patients with MetS. In our study, there was no significant difference observed in FoxO1 expression between the two groups among patients diagnosed with AV. However, our study revealed a remarkable rise in mTOR expression among AV patients with MetS in comparison with those without MetS. The mTOR pathway is known to be associated with metabolic diseases [30], confirming the data of our study.

miRs are involved in several steps of AV etiopathogenesis. miR-21 has pro-inflammatory effects and is also associated with the fibrotic process in patients with AV [12]. In the study by Ghumra et al. [1], it was shown that miR-21 levels were increased in both the plasma and tissue of patients with AV and that this miR was an early marker for the development of atrophic scarring. In our study, miR-21 level was found to be higher in patients with MetS accompanied by AV. This suggests that miR-21 is associated with the inflammatory process of AV.

miR-29b increases serum branched-chain amino acids, thereby activating the mTORC1 pathway (Figure 6) [27]. In the study by Ghumra et al [1], it was observed that miR-29b expression was not altered in patients with AV compared with the normal population. In our study, miR-29b levels were found to be higher in patients with MetS accompanied by AV. Therefore, we suggest that miR-29b, a member of a diabetogenic family, may have a significant impact on the pathogenesis of AV patients with MetS.

In their study, Khan et al. [30] established that apoptosis was inhibited and cell proliferation was increased in immortalized human keratinocyte cells when circulating miR-98 levels decreased in diabetes, and skin changes secondary to diabetes were associated with this process. After conducting a review of the available literature, we did not find any study that investigated the level of miR-98 in patients with AV. 

Our study has the following limitations: 1) the small size of the patient and control groups, 2) limited generalizability of the results, since it was conducted only on Turkish patients, 3) the impossibility of determining the levels of FoxO1, mTOR, miR-21, miR-29b, and miR-98 in plasma samples, and 4) while the expression levels of target genes, indicative of the functional analysis of FoxO1 and mTOR, were examined, the phosphorylation status of FoxO1 and mTOR, as well as the total protein levels of FoxO1 and mTOR, were not investigated.

## Conclusions

Acne vulgaris patients without MetS were found to be younger in this study. In all three groups, the male gender was dominant. Plasma HDL-C levels were lower in AV patients with MetS than in AV patients without MetS or the healthy control group. No difference was detected between the groups when AV severity was evaluated according to the GAGS scores. HOMA values were higher in patients with MetS. An examination of HOMA values in AV patients with MetS revealed that those with moderate GAGS scores had higher HOMA values than those with mild GAGS scores.

FoxO1 expression was decreased in both AV patients with MetS and AV patients without MetS. Thus, the importance of the FoxO1 signaling pathway in the pathogenesis of AV was once again demonstrated. Furthermore, it was found that mTOR gene expression levels were increased in AV patients with MetS, and it was demonstrated that the mTOR pathway plays an important role, especially in AV patients with MetS.

The levels of miR-21 and miR-29b were higher in AV patients with MetS. These data indicate that miR-21 and miR-29b are linked to the inflammatory process of AV.
